# The Dark Triad and Sexual Assertiveness Predict Sexual Coercion Differently in Men and Women

**DOI:** 10.1177/0886260520922346

**Published:** 2020-05-21

**Authors:** Minna Lyons, Emma Houghton, Gayle Brewer, Freya O’Brien

**Affiliations:** 1 University of Liverpool, UK

**Keywords:** Dark Triad traits, narcissism, Machiavellianism, psychopathy, sexual assertiveness, sexual coercion

## Abstract

Sexual coercion is a global problem that has been studied widely with regard to various characteristics of the perpetrators. The Dark Triad of personality (i.e., narcissism, Machiavellianism, and primary and secondary psychopathy) has been indicated as an important predictor of coercive cognitions and behaviors. In this study, we report findings of an online study (*N* = 208), exploring the relationship between sexual coercion, the Dark Triad, and sexual assertiveness (i.e., strategies for achieving sexual autonomy). We found that the Dark Triad was a stronger predictor of sexual coercion in men than in women. In men, all the Dark Triad components were significantly, positively correlated with sexual coercion, and narcissism and Machiavellianism had significant, negative correlations with sexual assertiveness. In women, only narcissism had a significant, positive correlation with sexual coercion, and the Dark Triad traits were not correlated with sexual assertiveness. In regression analyses, controlling for shared variance between the predictor variables, high secondary psychopathy, and low sexual assertiveness emerged as significant predictors of coercion in men. Only narcissism was a significant positive predictor in women. We discuss the results with a reference to evolutionary Life History theory.

## Introduction

Sexual coercion may be defined as “the act of using pressure, alcohol or drugs, or force to have sexual contact with someone against his or her will” (Struckman et al., 2003, p.76). It is an important global issue that occurs both in established relationships and outside this context (Shackelford & Goetz, 2004) and has a range of negative consequences for those involved (Oram et al., 2017; Stermac et al., 2018). Numerous individual predictors of sexual coercion perpetration have been identified, including sex (i.e., being male; Struckman et al., 2003), high impulsivity (Wilhite & Fromme, 2017), and high sexual promiscuity, trait aggression, and low empathy (DeGue et al., 2010). In the present study, we extend this research to investigate the role of sex, socially aversive personality traits (i.e., the Dark Triad), and sexual assertiveness in the perpetration of sexual coercion.

The Dark Triad (narcissism, Machiavellianism, and psychopathy) is a widely studied constellation of socially malevolent personality traits, characterized by behaviors and cognitions also typical to perpetrators of sexual coercion. In a similar manner to perpetrators, high levels of the Dark Triad relate to impulsivity (Malesza & Ostaszewski, 2016), aggression (Jones & Neria, 2015), poor self-control (Jonason & Tost, 2010), low empathy (Jonason et al., 2013), and promiscuous mating (Koladich & Atkinson, 2016). The Dark Triad constellation has been conceptualized as a manifestation of a fast life history strategy, where the reproductive focus is on the acquisition of short-term mating opportunities with multiple partners rather than investing in long-term, stable partnerships (Jonason et al., 2010). Indeed, previous research has associated both fast life history strategy (Gladden et al., 2008; Kavish & Anderson, 2019) and the Dark Triad (Figueredo et al., 2015) with sexually coercive behavior. It is possible that exploitative sexual tactics such as sexual coercion have evolved as part of a fast life history strategy, with the Dark Triad traits facilitating this strategic approach.

Fast life history strategy is more closely associated with male than female sexual behavior. This is consistent with previous research indicating that although women’s perpetration is not rare (Stemple et al., 2017; Weare, 2018), men are significantly more likely than women to coerce (Fernández-Fuertes et al., 2018; Tomaszewska & Krahé, 2018). Consistent with reports that the Dark Triad traits facilitate a short-term mating strategy in men, previous research also indicates that Dark Triad traits are higher in men than women, and exert a greater influence on male compared with female behavior (Jonason et al., 2009; Muris et al., 2017; Paulhus & Williams, 2002). Indeed, the impact of specific Dark Triad traits may differ for men and women. For example, aspects of psychopathy are more strongly associated with low empathy in men than in women (Jonason et al., 2013). It has been suggested that men may be more sexually coercive than women because of higher levels of Dark Triad traits (Jonason et al., 2017).

There is a considerable body of research relating Dark Triad traits to sexually exploitive cognitions, intentions, and behaviors. Those high on the Dark Triad traits have a tendency to accept rape myths (Jonason et al., 2017), blame victims of sexual aggression (Brewer et al., 2019), and endorse a hostile environment conducive to sexual harassment (Zeigler-Hill et al., 2016). Psychopathy appears to be the strongest predictor of these cognitions. With regard to coercive behavior, both psychopathy (Jones & Olderbak, 2014; Muñoz et al., 2011) and narcissism (Blinkhorn et al., 2015) predict the self-reported use of coercion after rejection of sexual advances. While some studies highlight the relationship between primary psychopathy (i.e., cold, callous, interpersonal-affective) and sexual coercion (Brewer et al., 2019; Muñoz et al., 2011), others suggest that aspects of secondary psychopathy (i.e., impulsive, antisocial behavior) predict coercion reflecting an increased motivation to feel powerful (Hoffmann & Verona, 2019). Indeed, there may be many motivations, cognitions, and strategies that connect the Dark Triad to sexual coercion.

One aspect of sexual behavior that could be related to both coercion and the Dark Triad is sexual assertiveness. Sexual assertiveness consists of strategies that individuals use to achieve sexual autonomy (Hurlbert, 1991). Sexually assertive behavior includes openly talking about one’s sexual desires, taking an active role in initiating sex, as well as the ability to refuse the undesired sexual advances of others. Higher assertiveness has been associated with multiple positive outcomes, such as better functioning relationships (Greene & Faulkner, 2005), a more satisfying sex life (Haavio-Mannila & Kontula, 1997), and a reduced likelihood of being a victim of sexual coercion (Kelley et al., 2016) or sexual assault (Franz et al., 2016). Although relatively few studies have investigated the sexual assertiveness of perpetrators, researchers report that higher perpetrator assertiveness is related to recognition of sexual consent in complex scenarios (Shafer et al., 2018) and lower perpetration of sexual aggression by young women (Krahé et al., 2015). Thus, it may be expected that higher assertiveness is correlated with less frequent use of coercive mating tactics.

Interestingly, although the Dark Triad is connected to promiscuous, noncommitted sexual lifestyles and interpersonal aggression, those high in these traits (especially Machiavellianism and secondary psychopathy) are not necessarily sexually assertive. Indeed, while high narcissism is associated with greater sexual assertiveness, those high in psychopathy and women high on Machiavellianism are less sexually assertive (Pilch & Smolorz, 2019). Sexual assertiveness does not seem to be a prerequisite for fast life history strategy. Furthermore, in women, sexual assertiveness may even be a protective factor against compliance with unwanted sex and sexual victimization (Darden et al., 2019). It is, therefore, important to consider the relative influence of both Dark Triad traits and sexual assertiveness on perpetration of sexual coercion by men and women.

The present study investigates the relative influence of sex, socially aversive personality traits, and sexual assertiveness on sexual coercion perpetration. We predict greater perpetration of sexual coercion by men compared with women and that Dark Triad traits will have a greater influence on men’s coercive behavior than women’s. Furthermore, we predict that sexual assertiveness will be associated with lower levels of sexual coercion perpetration.

## Method

### Participants

Men (*n* = 79) and women (*n* = 129) aged 18 to 69 years (*M* = 22.56, *SD* = 7.20; 49% single, 30% in a serious relationship, 19% dating casually, 2% preferred not to reveal their relationship status) completed an online “Personality and Sexual Behaviour” study. Participants were recruited via a British University participation point scheme and social networking sites.

Participants completed initial demographic questions online followed by a series of standardized self-report questionnaires. Narcissism (Cronbach’s α = .88) was measured with the 16-item Narcissistic Personality Inventory (NPI-16, Ames et al., 2005). Each of the 16 statement pairs contains a narcissistic (e.g., “I am an extraordinary person”) and nonnarcissistic response (e.g., “It makes me uncomfortable to be the center of attention”). Participants select the response which most applies to them. Narcissistic and nonnarcissistic responses were scored as 1 and 0, respectively.

Machiavellianism (α = .80) was measured with the 20-item Mach-IV (Christie & Geis, 1970) Participants respond to each statement (e.g., “Never tell anyone the real reason you did something unless it is useful to do so”) on a 7-point Likert-type scale (1 = *strongly disagree* to 7 = *strongly agree*). Ten items were reversed scored such that higher score indicate higher levels Machiavellianism.

Primary (α = .90) and secondary (α = .71) psychopathy were measured with the 26-item Levenson Self-Report Psychopathy Scale (Levenson et al., 1995). The measure contains 16 items for primary (e.g., “I enjoy manipulating other people’s feelings”) and 10 items for secondary (e.g., “Love is overrated”) psychopathy. Participants respond to items on a 5-point Likert-type scale (1 = *strongly disagree* to 5 = *strongly agree*) with seven items reverse scored such that higher scores indicate higher primary and secondary psychopathy.

Sexual Assertiveness (α = .71) was measured with the 25-item Hulbert Index of Sexual Assertiveness (HISA, Hurlbert, 1991). This original version was developed to be a one-dimensional scale of measuring sexual assertiveness in couples (Santos-Iglesias et al., 2014). Items mostly address the ability to initiate and communicate about desired sex (e.g., “I approach my partner for sex when I desire it”), but also examine the ability to refuse unwanted sex (e.g., “I find myself having sex when I do not really want it”), and communicate sexual history and risk (e.g., “I try to avoid discussing the subject of sex”). Participants rate each statement on a 5-point scale (0 = *never*, 1 = *rarely*, 2 = *some of the time*, 3 = *most of the time*, 4 = *all of the time*) with 12 items reverse scored such that higher scores indicate higher sexual assertiveness. As the original, one-dimensional version of the scale was used, the overall Cronbach’s alpha score was used to measure internal reliability.

Sexual coercion (α = .86) was measured with the19-item Postrefusal Sexual Persistence Scale (Struckman-Johnson et al., 2003). The scale measures engagement in sexually coercive tactics after sexual advances have been rejected. Participants indicate the frequency with which they have used the tactics on a member of the opposite sex since the age of 16 (0 = never experienced, 1 = 1–2 times, 2 = 3–5 times, 3 = 6–8 times, 4 = more than 8 times). Items address sexual arousal (e.g., “Continued to kiss or touch them aroused them”); emotional manipulation and deception (e.g., “Questioned their sexuality”); exploitation of the intoxicated (e.g., “Took advantage of the fact that they were already drunk or high”); and physical force, threats, and harm (e.g., “Tied them up”). Items were totalled to create an overall measure of sexual coercion.

## Results

Men scored higher than women in narcissism, *t*(1,206) = −7.98, *p* < .001, Machiavellianism, *t*(1,206) = −5.30, *p* < .001, primary, *t*(1,206) = −8.33, *p* < .001, and secondary, *t*(1,206) = −4.42, *p* < .001, psychopathy. Men also scored higher in perpetration of sexual coercion strategies, *t*(1,206) = −7.02, *p* < .001, but not in sexual assertiveness, *t*(1,206) = 0.71, *p* = .48. A Bonferroni correction was applied as multiple comparisons were examined, resulting in a significance threshold of 0.002. Pearson’s correlations indicate that, in men, narcissism, Machiavellianism, primary psychopathy, and secondary psychopathy were all significantly, positively correlated with sexual coercion. Moreover, narcissism and Machiavellianism had a significant, negative correlation with sexual assertiveness. In women, only narcissism had a significant, positive correlation with sexual coercion scores. In women, Dark Triad traits were not correlated with sexual assertiveness. Descriptive statistics and correlations are shown in Table 1.

**Table 1.** Descriptive Statistics and Pearson’s Correlations Between Dark Triad Traits, Sexual Assertiveness, and Sexual Coercion.

Variables	*M* (*SD*): Men	*M* (*SD*): Women	MA	NA	PP	SP	SA	SC
MA	62.40 (10.67)	55.10 (9.01)		.69*	.71*	.44*	–.37*	.60*
NA	8.38 (4.53)	3.88 (3.52)	.36*		.72*	.50*	–.43*	.62*
PP	3.91 (1.12)	2.73 (0.89)	.60*	.59*		.51*	–.27	.56*
SP	4.16 (1.06)	3.57 (0.88)	.46*	.25	.46*		–.14	.49*
SA	65.56 (18.46)	67.17 (14.34)	–.02	–.06	–.06	.01		–.51*
SC	10.63 (9.57)	3.33 (5.42)	.16	.30*	.25	.19	–.09	

*Note.* Men above and women below the diagonal. MA = Machiavellianism; NA = narcissism; PP = primary psychopathy; SP = secondary psychopathy; SA = sexual assertiveness; SC = sexual coercion.

*p < .00.

A series of Fisher’s *r* to *z* transformations were conducted, using the online vassarstats.net calculator, to assess correlation strength sex differences. There were significant sex differences in the correlations between all Dark Triad traits and sexual coercion (Fisher’s zs = 2.30–3.66, all *p*s < .001), demonstrating that the Dark Triad is a stronger predictor of coercion in men than it is in women. Correlations between sexual assertiveness and sexual coercion were significantly different for men and women, indicating a stronger, negative relationship in men (Fisher’s *z* −.3.30, *p* < .001).

We conducted multiple linear regressions separately for each sex to investigate the relative influence of sexual assertiveness and the Dark Triad in self-reported sexual coercion (see Table 2). At Step 1, we entered sexual assertiveness, and at Step 2, narcissism, Machiavellianism, primary psychopathy, and secondary psychopathy were included. In men, the Step 1 model was significant, *F*(1, 77) = 27.37, *p* = .001, explaining 25% of variability in sexual coercion. Sexual assertiveness was a significant negative predictor (β = −.51, *t* = −5.23, *p* = .001) of sexual coercion. Adding the Dark Triad at Step 2 improved the model, *F*^2^ change (4, 73) = 11.55, *p* = .001. Together, sexual assertiveness and the Dark Triad explained 55% of variability in sexual coercion scores in men. When all five predictors were entered into the model, sexual assertiveness remained a significant negative predictor, although to a lesser degree (β = −.31, *t* = −4.47, *p* = .001). Of the Dark Triad trait variables, only secondary psychopathy was a significant positive predictor of coercion (β = .22, *t* = 2.36, *p* = .021). Men low on sexual assertiveness and high on secondary psychopathy were more likely to engage in sexual coercion.

**Table 2.** Multiple Regression Showing the Predictive Ability of Sexual Assertiveness and Dark Triad on Self-Reported Sexual Coercion.

Steps	Predictor	Unstandardized Coefficient		Standardized Coefficient		*R* ^2^	Δ*R*^2^	*F*	Δ*F*
		*B*	*SE*	β	*p*				
Men									
1	–	–	–	–	–	.26	_	27.37**	–
	Constant	28.02	3.45						
	SA	–0.27	0.05	–.51	.00				
2	–	–	–	–	–	.55	.27	17.70**	11.55**
	Constant	–4.89	6.72	–	–				
	SA	–0.16	0.05	–.31	.00				
	NA	0.36	0.28	.17	.19				
	MACH	0.17	0.11	.19	.12				
	PP	0.93	1.10	.11	.40				
	SP	2.09	0.89	.22	.02				
Women									
1	–	–	–	–	–	.01	–	0.95	–
	Constant	5.52	2.30	–	–				
	SA	–0.03	0.03	–.86	.33				
2	–	–	–	–	–	.11	.10	3.02*	3.52**
	Constant	0.66	3.72	–	–				
	SA	–0.03	0.03	–.07	.44				
	NA	0.35	0.16	.23	.03				
	MACH	–0.01	0.07	–.01	.09				
	PP	0.42	0.77	.07	.59				
	SP	0.65	0.61	.11	.29				

*Note.* NA = narcissism; PP = primary psychopathy; SP = secondary psychopathy; SA = sexual assertiveness; MACH = Machiavellianism.

*p < .05. **p < .001.

In women, sexual assertiveness did not predict sexual coercion at Step 1, *F* (1, 127) = 0.95, *p* = .331. Including the Dark Triad traits at Step 2 improved the model, *F*^2^ change (4, 123) = 3.52, *p* = .009. Together, sexual assertiveness and the Dark Triad traits explained 7.3% of variability in women’s sexual coercion scores. Only narcissism was a significant positive predictor of coercion (β = .23, *t* = 2.18, *p* = .031), such that women high on narcissism are most likely to engage in sexual coercion.

## Discussion

Our study sought to understand how sex, socially aversive personality traits, and sexual assertiveness relate to self-reported sexual coercion. Consistent with initial predictions, men reported higher levels of sexual coercion than women, and the Dark Triad traits were more closely associated with the coercion perpetrated by men. The results support previous studies that have found that women possess slow life history traits that act as a protective factor against perpetration of sexual coercion (Gladden et al., 2008). Theoretically, women may gain more evolutionary benefits not from coercive or promiscuous mating, but from manipulating and controlling partners within a longer term relationship context (Moore et al., 2020). Thus, the fast life history strategies in high Dark Triad women may be enacted via manipulation in serial/simultaneous relationships (see also Brewer & Abell, 2017; Brewer et al., 2018), rather than via sexual coercion of partners. Men, in turn, may benefit more from strategies that increase the number of matings and sexual partners (Moore et al., 2020), influencing the predisposition of sexual coercion in high Dark Triad men.

With regard to the Dark Triad traits, men high in psychopathy were more likely to report perpetration of sexual coercion, which is consistent with previous research (e.g., Hersh & Gray-Little, 1998). Indeed, some researchers describe coercive sexuality as a fundamental aspect of psychopathy (Harris et al., 2007). Although primary psychopathy is often most closely associated with sexual coercion (Wiebe, 2004), secondary rather than primary psychopathy predicted perpetration in the present study. Secondary psychopathy is characterized by impulsive, antisocial behavior, and social deviance, and research indicates that men high on impulsivity are more likely to endorse sexually predatory behavior (O’Connell & Marcus, 2016) and engage in relational aggression (Czar et al., 2011). Hence, greater sexual coercion may, in part, reflect lower levels of self-control.

Interestingly, in our study, women (but not men) who were high in narcissism used more sexually coercive strategies. This is consistent with previous research reporting that narcissism, and maladaptive facets of narcissism in particular, is associated with increased sexual coercion in women (Blinkhorn et al., 2015; Ryan et al., 2008). Women high in narcissism are more likely to engage mate poaching for both casual and long-term romantic relationships, suggesting greater sexual aggression and motivation (Brunell et al., 2018). Furthermore, within established relationships, narcissistic individuals try to influence their partner by using bullying and manipulation (Sauls et al., 2019). The increased use of aggression and coercion may reflect the tendency to engage in sexual activity to fulfill the need for self-affirmation (Gewirtz-Meydan, 2017). Together, our findings and previous literature point at the willingness of highly narcissistic women to resort to socially frowned upon methods in pursuing what they want, both within casual and serious relationships.

Contrary to predictions and previous research (Krahé et al., 2015), sexual assertiveness did not predict women’s perpetration of sexual coercion. As Krahé et al. (2015) noted, the relationship between assertiveness and coercion is associated with several country-level variables, including male–female relations in a given culture. Although we do not have exact data for the location of our sample, our participants were likely to be mostly from the United Kingdom. Krahé et al. (2015) examined data from 10 European countries (excluding the United Kingdom), finding significant between-country differences in sexual assertiveness and coercion. Future studies should investigate whether there are any aspects of the U.K. culture that could explain the lack of relationship between assertiveness and coercion in women in our sample.

For men, however, sexual assertiveness was associated with lower perpetration of sexual coercion. In part, this may reflect greater sexual confidence displayed by sexually assertive men and willingness to discuss sexual issues. For example, open communication (including a willingness to reject their partner’s advances) may result in sexually assertive men being less sensitive or offended when rejected themselves. For example, they may have a greater appreciation of variation in sexual receptivity and feel more secure in their relationship. Alternatively, sexually assertive men may have a greater appreciation of sexual consent and be more sensitive to consent cues (Shafer et al., 2018). As few relatively few studies have considered assertiveness in relation to perpetration, additional research is needed to explore this area.

### Limitations and Future Research

One of the criticisms for our study, and the Dark Triad research as a whole, is the lack of diversity in the study populations (Lyons, 2019). Like majority of the Dark Triad studies, we had a limited internet sample of British University students and participants recruited through researchers (mainly the United Kingdom) social media contacts. Future research should investigate how the Dark Triad and assertiveness relate to coercion beyond Western, Educated, Industrial, Rich, and Democratic samples (i.e., WEIRD; Henrich et al., 2010). The Dark Triad exerts its influence on interpersonal behaviors differently depending on the cultural context (Robertson et al., 2016)

Furthermore, subsequent studies may incorporate measures of self-rated mate value. It has been suggested that perceived competitive disadvantage serves as a pathway to sexual exploitation and aggression (Lalumière et al., 2005; Nunes & Pettersen, 2011). Specifically, fast life history strategies in men could be geared toward sexual coercion via low self-perceived mate value (Gladden et al., 2008). It is possible that those who are low in self-perceived mate value become more sexually assertive when interacting with partners and this becomes a pathway to coercive strategies. This research should particularly consider coercion directed both within an existing relationship and outside this context.

To conclude, we investigated sex, socially aversive personality traits, and sexual assertiveness in relation to self-reported sexual coercion. Men reported higher levels of Dark Triad traits and sexual coercion perpetration than women, but not higher sexual assertiveness. Furthermore, Dark Triad traits and sexual assertiveness were more closely associated with men’s use of coercion than women’s coercive behavior. Findings are broadly consistent with the suggestion that sexual coercion may support a male fast life history strategy that focuses on present rather than future-oriented reproductive goals, facilitated by Dark Triad traits. Additional research is required to further explore the impact of sexual assertiveness on sexual behavior and in reducing the incidence of sexual coercion perpetration in particular.
